# Identification of End-User Economical Relationship Graph Using Lightweight Blockchain-Based BERT Model

**DOI:** 10.1155/2022/6546913

**Published:** 2022-05-06

**Authors:** Mukta Jagdish, Devangkumar Umakant Shah, Varsha Agarwal, Ganesh Babu Loganathan, Abdullah Alqahtani, Saima Ahmed Rahin

**Affiliations:** ^1^Department of Information Technology, Vardhaman College of Engineering (Autonomous), Hyderabad, Telangana, India; ^2^Department of Electrical Engineering, K. J. Institute of Engineering & Technology, Savli, Vadodara, India; ^3^Center for Management Studies, Jain (Deemed-to-be-University), Bangalore, India; ^4^Department of Mechatronics, Faculty of Engineering, Tishk International University-Erbil, Kurdistan Region, Iraq; ^5^Department of Computer Science, College of Computer Science, King Khalid University, Abha, Saudi Arabia; ^6^United International University, Dhaka, Bangladesh

## Abstract

Current methods for extracting information from user resumes do not work well with unstructured user resumes in economic announcements, and they do not work well with documents that have the same users in them. Unstructured user information is turned into structured user information templates in this study. It also proposes a way to build person relationship graphs in the field of economics. First, the lightweight blockchain-based BERT model (B-BERT) is trained. The learned B-BERT pretraining model is then utilized to get the event instance vector, categorize it appropriately, and populate the hierarchical user information templates with accurate user characteristics. The aim of this research is that it has investigated the approach of creating character connection graphs in the Chinese financial system and suggests a framework for doing so in the economic sector. Furthermore, the relationship between users is found through the filled-in user information template, and a graph of user relationships is made. This is how it works: finally, the experiment is checked by filling in a manually annotated dataset. In tests, the method can be used to get text information from unstructured economic user resumes and build a relationship map of people in the financial field. The experimental results show that the proposed approach is capable of efficiently retrieving information from unstructured financial personnel resume text and generating a character relationship graph in the economic sphere.

## 1. Introduction

In the information age, companies and regulatory agencies in the financial industry publish many announcements on the internet every day, extract the personnel entities and related attributes in the reports, discover the implicit relationship between personnel, and describe the form in a structured way. Concepts, entities, and their relationships related to people in the financial industry build a relationship map of people in the economic field, conduct an in-depth analysis of financial activities, help financial practitioners make vital decisions such as partner selection and personnel appointment, and promote financial activities. It is essential to smoothly do it. In addition, with the rapid development of our country's economy, financial activities are becoming more and more frequent, and economic crimes are more and more difficult to detect and supervise. By constructing a relationship graph of people in the financial industry, social relationships such as alumni and colleagues of financial practitioners can be found, and early warnings can be provided. Potential financial activity risks and the fight against economic crime are also significant. The key to building a personnel relationship graph in the financial sector is extracting personnel-related entities, attributes, and events from unstructured economic announcements, which entails tasks such as named entity recognition [1, 2], relationship extraction, and event extraction [3]. In recent years, as computer power has increased, deep learning technology based on neural networks has increasingly become the de facto standard way for named entity recognition. The neural network-based approach views named entity recognition as a sequence labeling task, constructs a multilayer neural network model from the text, represents words or characters in the text as word vectors, and uses them as model input to classify words or characters using neural networks—network model. The recurrent neural network (RNN) is an artificial intelligence system that works with consecutive or time series analysis. A convolutional neural network (CNN) is a deep neural network that is especially built to interpret pixel input and is used in pattern recognition systems. The convolutional neural networks are unable to adequately understand temporal data. From the text, named entities are extracted. Convolutional neural networks (CNN) [4], recurrent neural networks (RNN) [5], and others are examples of standard neural networks. The author [6] developed a range of sequence annotation models based on long short-term memory (LSTM) networks. Comparative investigations demonstrate that the bidirectional LSTM-connected conditional random field (CRF) model outperforms the LSTM-connected conditional random field (CRF) model.

Named Entity Recognition Effects: In this approach, the neural network-based technique builds a multilayer neural network model from the text, representing words or characters as word vectors and employing them as model input to classify words or characters using neural networks. Author [7] uses dilated convolutions for named entity recognition, which increases the receptive field and improves the training and prediction speed of the model; pretrained language models [8] can learn latent semantic information from a large number of unlabeled texts for downstream natural language processing tasks that provide better feature representations [9]. The proposed method is more beneficial than the previous method in respect of its accuracy. A light-weighted blockchain BERT model is used to correctly extract personnel attribute entities, and the trained BERT model is used to classify event instance vectors, construct a hierarchical personnel information template, and solve the problem of personnel attribute association. The BERT [10] pretrained language model achieves the best results on 11 natural language processing tasks, and applying BERT to the named entity recognition task can achieve better results. Entity relation extraction refers to extracting predefined entity relations from unstructured text based on entity recognition. Traditional relation extraction [11, 12] finds the connection between entities in a sentence, and most of them do not further extract the attributes of the relation.

They cannot find the link between people across documents in financial announcements. A person as an entity often contains several entity attributes (such as date of birth and gender) [13]. Character attribute extraction establishes the relationship between characters and entity attributes, which can be regarded as a particular form of relation extraction. There are few studies on extracting structured person attributes from unstructured person texts. Author [14] used Wikipedia data describing people as corpus input and output structured people containing only work experience by analyzing sentence dependency graphs. Resume Information: author [15] uses Wikipedia and Wikidata as data sources to extract information about people who meet specific requirements but involves relatively few attributes of people.

Unstructured personnel resume text usually contains multiple employment events and education events. Accurately extracting numerous employment events and educational events of personnel resumes without trigger words is a problem worthy of study. The CRFs are a type of statistical modeling tool used for organized prediction in object detection and recognition. Comparative investigations demonstrate that the bidirectional LSTM-connected conditional random field (CRF) model outperforms the LSTM-connected conditional random field (CRF) model. Existing event extraction methods [16, 17], usually for news and other corpora, mainly rely on trigger words to detect certain events and then extract relevant event parameters, which are not suitable for unstructured personnel resume texts [18]. Author [19] proposed that event types can be detected through the critical parameters in the event, without relying on trigger words to see possibilities and extract event parameters. Still, it cannot solve the problem of unstructured personnel resumes with multiple employment events and education events' special circumstances.

Aiming at the unique situation of unstructured personnel resumes, this study studies how to fill hierarchical personnel information templates that extracts the relationship between personnel across documents. It removes multiple positions in the unstructured personnel resume text without relying on trigger words. Furthermore, experience and educational experience events, a BERT-based framework for constructing a relationship graph of Chinese characters in the financial field, are proposed. Experiments demonstrate that the suggested technique is capable of efficiently resolving the problem of extracting information from unstructured financial personnel resume text and constructing a relationship graph of characters in the economic area.

This study has been planned into various sections.  Section 1 dealt with introducing the concept and importance of the BERT model.  Section 2 puts light on related works. The construction framework of the relationship graph has been mentioned in Section 3. The experiment and analysis have been described in Section 4. Finally, Section 5 portrays the conclusion and possible future works based on the proposed framework.

## 2. Related Work

**Figure 1:**
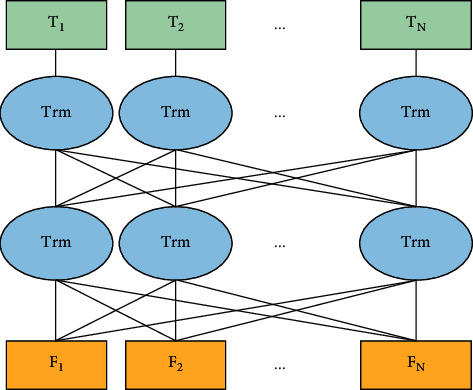
The BERT model structure.

The BERT is a Google-developed pretrained language model that got the top results in 11 natural language processing tasks. It represents one of the most important improvements in natural language processing in recent years. The masked language model masks some words at irregular intervals by uniformly substituting some words with identifiers and afterward assumes these masked words using the context information of the masked terms, allowing the vector representation of every word to relate to relevant information in a clear and concise manner. BERT is a paradigm for deep bidirectional language representation based on transformer [20]. The transformer design is used to produce a multilayer bidirectional encoder network. The fundamental structure is seen in Figure 1. *F* denotes the word that corresponds to each word in the input sentence. The vector, Trm, denotes the transformer encoder, and *T* is the output word vector for each word in the input phrase.

The input word vector of the BERT model is obtained by adding three parts: token embedding, segment embedding, and position embedding. The word representation represents the initial word vector of the current word, which is usually obtained by looking up a table; the segment representation represents which sentence the present word belongs to; the position representation represents the position index of the current term in the sentence. In addition, the original input of the sentence needs to add (CLS) and (SEP) tags; (CLS) is added at the beginning, which can be used to represent the entire sentence; the (SEP) tag is used to separate two sentences, indicating the end of the sentence.

The BERT pretraining process consists of two different pretraining tasks, the masked language model and the following sentence prediction task. First, the masking language model randomly masks some words by uniformly replacing some words with identifiers (MASK) and then predicts these masked words by the context information of the masked terms so that the vector representation of each word can comprehensively refer to contextual information [21]. Next sentence prediction refers to predicting whether a particular sentence is the following sentence of another sentence. In this way, the relationship between sentences is introduced into the model to obtain semantic information between sentences.

After the pretraining of the BERT model is completed, the model parameters in the pretraining process are adjusted by fine-tuning and retraining so that the model is more suitable for downstream tasks to obtain better results. The event instance vector is obtained using the learned B-BERT pretraining model, which is then suitably classified and used to populate the hierarchical user information templates with accurate user attributes. The B-BERT pretraining model is used to classify event instance vectors, construct a hierarchical personnel information template, and solve the problem of personnel attribute association.

For example, for sentence-level classification tasks, the output vector representation of the first label (CLS) is taken as the sentence representation; for character-level classification tasks, the output of the last layer transformer of all characters is taken and sent to the softmax layer for classification.

## 3. Construction Framework of the Relationship Graph

The BERT pretrained language model is used to build a personal relationship graph in the financial field, and the personal relationship graph is used to create a framework. The BERT-Template technique is used to fill the personnel template, which solves the problem of personnel attribute association. The personnel attribute entity is extracted from the unstructured personnel resume text using the prediction findings. The framework is divided into three parts: the first part is personnel attribute entity extraction, which uses the BERT model to extract personnel attribute entities such as the birth date and employer from the resume text of financial personnel; the second part is personnel attribute association, which is defined and analyzed by fill in the personnel template, associates the personnel attribute name with the personnel attribute value, and associates the relevant personnel attribute value to form a job event or education event; the third part is the construction of the personal relationship graph, which uses the personnel template to discover the relationship between personnel. Finally, the character relationship graph storage model is defined and the graph database is used to store the character relationship graph.

### 3.1. Person Attribute Entity Extraction Based on BERT

The model consists of an input layer and 24 hidden layers. The output of the last hidden layer is the vector representation of each character corresponding to each character [22]. The vector representation of each character is used to carry out personnel attributes.

Classification of entities inputs the vector encoding of the character into the linear classifier, and then, the softmax operation is gone through to obtain the probability distribution of each symbol corresponding to each personnel attribute label, and the personnel attribute label corresponding to the maximum probability value as the final personnel attribute label of the current character classification is selected [23]. After obtaining all characters' model-predicted personnel attribute classification, the prediction results are processed to obtain the personnel attribute entity in the unstructured personnel resume text.

### 3.2. Person Attribute Association Based on BERT

The personnel attributes (such as date of birth, personnel position, and company) extracted from the BERT model may have multiple candidate values. Therefore, it is necessary to determine the personnel attribute values that uniquely correspond to some personnel attribute names to associate them with the personnel attribute values associated. In addition, there is an association relationship between some personnel attribute values, and the personnel attribute values with the association relationship constitute an event instance.

For example, attribute values such as tenure time, resignation time, tenure unit, tenure department, and position form a tenure event instance. Therefore, it is necessary to correctly associate and combine relevant attribute values to identify and filter out the correct event instance [24]. The personnel attribute association task is solved by populating the personnel template through the BERT-Template method. Person template consists of fixed key-value (key-value) pairs stored in JSON file format; personnel entities are described in a structured form; and personnel attribute information is recorded.

The key of the personnel template is used to identify the attribute of the personnel, which is usually represented by a string; the value of the personnel template corresponds to a specific key, which can be an array or a particular value. A legend whose value is an array is called a multivalued attribute of the person template, and a key whose value is not an array is called a single-valued attribute of the person template [25]. The single-valued attribute of the personnel template associates the personnel attribute with the personnel attribute entity. For the single-valued attribute of the personnel template, a specific strategy is used to fill it. Usually, the personnel attribute entity with the most occurrences corresponding to the single-valued attribute is selected for filling [26, 27]. The multivalue attribute of the personnel template records a list of event instances and associates the personnel attribute entities involved in the event.

**Figure 2:**
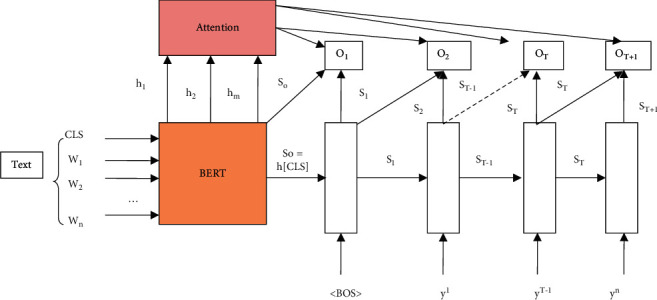
Structure diagram of an event classification model.

The core of completing the task of personnel attribute association is to establish the association between the multivalued attribute entities of personnel. The multivalued attributes of the personnel template are filled in through the BERT-Template method to complete the association between the multivalued attribute entities of the personnel [27, 28]. This method classifies and judges the authenticity of the event instance by obtaining the event instance vector to not rely on the trigger word to extract the multivalued personal, educational, and employment experience events [29, 30]. The model architecture for event instance classification is shown in Figure 2.

(1) $$\begin{array}{c} Q=W_{1}⊕W_{2}⊕\cdots ⊕W_{i}⊕\cdots ⊕W_{n}\\ W_{i}=\text{MaxPooling}\left(fw_{i}\right). \end{array}$$

In the formula, *Q* represents the final event instance vector, *V*_*i*_ represents the character attribute entity vector, **fw**_*i*_ represents the feature vector group of the character attribute entity, MaxPooling represents the maximum pooling operation, and ⊕ represents the vector splicing operation. First, the ultimate pooling operation (MaxPooling) is performed on the feature vector group of the person attribute entity to obtain the maximum value of each dimension of the vector in the vector group, and then, the total value of each size is combined into a new vector as the person attribute entity vector, the record is Wi; the maximum pooling operation is performed on the character vector group of all personnel attribute entities of the current event instance, all personnel attribute entity vectors {*W*_1_, *W*_2_,…, *W*_*i*_,…, *W*_*n*_} of the current event instance are obtained, and all personnel attributes are spliced. The entity vector gets the final event instance vector *Q*. The last is the output layer of the model. The event instance vector is sent to a fully connected network for classification at the output layer to determine whether it is an actual event instance.

### 3.3. Character Relationship Diagram Construction

A person template contains a list of event instances consisting of single-valued person attributes and multivalued person attributes. Hierarchical people templates can be used to discover relationships between people entities. A certain multivalued attribute of the personnel template can be denoted as attr =  {*f*_1_, *f*_2_,…*f*_*i*_,…, *f*_*n*_*|n* ≥ 0}, where *f*_i_ represents the multivalued attribute of personnel, denoted as *f*_*i*_={*u*_1_, *u*_2_,…, *u*_*i*_,…*u*_*m*_*|m* ≥ 2}, where *u*_*i*_ is the attribute value of the person. The same multivalued attributes in personnel template B and personnel template *C* are denoted as attr_B and attr_C. A certain personnel attribute entity in personnel templates B and C is denoted as *u*_*B*_ and *u*_*C*_, respectively. If {*u*_*B*_=*u*_*C*_*|*(*u*_*B*_ ∈ *f*_*i*_ ∈ attr_*B*)∧(*t*_*C*_ ∈ *f*_*i*_ ∈ attr_*C*)} ≠ ∅, it can be considered that personnel template B and personnel template C have the assignment attribute *f*_*i*_ of co-occurrence relation personnel in multiple dimensions, namely, person B and person C have a certain relationship. When *f*_*i*_ is a graduate school, B and C are considered to have alumni; when *f*_*i*_ is a work unit, B and C are considered colleagues.

The person entity is mapped to the person node in the Neo4j graph database, the single-valued attribute of a person and its corresponding person attribute entity are mapped to the attribute key-value pair of the person node in the Neo4j graph database, the colleague relationship is mapped, and alumni relations in the Neo4j graph database are mapped as an edge.

After defining the data model of the personal relationship graph in the graph database, the information in the person template and the personal relationships is stored that is found in Section 3.3 in the Neo4j database.

## 4. Experiments and Analysis

### 4.1. Experimental Dataset

There is no public resume text dataset in the Chinese financial field. Therefore, this study crawled the annual reports, prospectuses, and documents on the official website of listed companies from the internet and obtained the unstructured personnel resume text information in the financial announcements. The unstructured personnel resume text information is labeled with BIO (B-begin, I-inside, and O-outside) related to personnel attribute entities through manual annotation. The basic knowledge of the personnel attribute entity annotation dataset is shown in Table 1.

A hierarchical personnel template is manually constructed, and several experts in the financial field are invited to proofread and modify the generated dataset to ensure the accuracy of the dataset. The basic information of the artificially constructed hierarchical personnel template dataset is shown in Table 2.

### 4.2. Hyperparameter Settings

This study uses albert_large_zh as the basic model, consisting of 24 transformer encoders, each transformer encoder contains 16 attention heads, and the dimension of the word vector is 1,024. In the fine-tuning training phase, the batch size is set to 32, the corresponding learning rate is set to 2E-5, the warm-up rate is set to 0.1, the maximum sentence length is set to 128, the dataset is iterated six times, and Adam is used for optimization on a RTX2080Ti machine training.

### 4.3. Person Attribute Entity Extraction Experiment and Result Analysis

**Figure 3:**
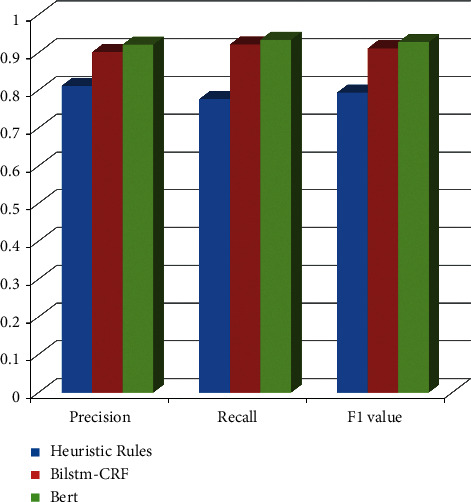
Confusion element result over personnel attribute entity extraction.

This study uses precision, recall, and *F*_1_ value as evaluation indicators for entity extraction of personnel attributes. They are compared with the method based on heuristic rules and the classic BiLSTM-CRF [6] method. The method based on heuristic rules extracts the attribute entities in the text by manually writing some templates or regular expressions. The experimental results of personnel attribute entity extraction are shown in Table 3 and Figure 3.

It can be seen from Table 3 that the BERT-based personnel attribute entity extraction method and the BiLSTM-CRF-based method all exceed 0.900 0 in the three evaluation indicators of precision, recall, and G1 value. Compared with the process based on BiLSTM-CRF, the BERT-based personnel attribute entity extraction method has achieved the best results in the three evaluation indicators of precision rate, recall rate, and *F*_1_ value. However, the heuristic rule method relies on manual regulations, and it is difficult for the authorities to cover all cases. Therefore, the precision and recall are the worst compared with the F1 value and the other two methods.

### 4.4. Experiment and Result Analysis of Personnel Attribute Association Method

After extracting the personnel attribute entity from the unstructured text, a hierarchical personnel template is constructed through the BERT-Template method to complete the personnel attribute association. The training data are divided into a training set and a test set according to the ratio of 9 : 1, and the training set is used for 8 000 iterations of training. The test set is used to test the model during the training process. The BERT model replaced the BiLSTM-CRF model as a comparative experiment.

The evaluation indicators of the final classification of educational experience event instances and employment experience event instances are shown in Table 4. The calculation method of accuracy is shown in equation (2), where TP represents the number pieces of correctly classified as a particular class, FP represents the number of pieces incorrectly classified as samples of the current category, FN represents the number of samples incorrectly classified as samples of other courses, and TN represents the number of antagonistic classes predicted as negative class numbers.


(2) $$\begin{array}{c} \text{Accuracy}=\frac{\text{UP}+\text{UN}}{\text{UP}+\text{GP}+\text{GN}+\text{UN}}. \end{array}$$

**Figure 4:**
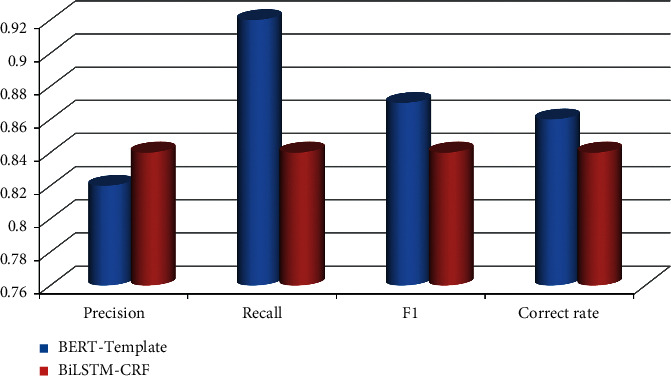
Event classification results on work experience.

**Figure 5:**
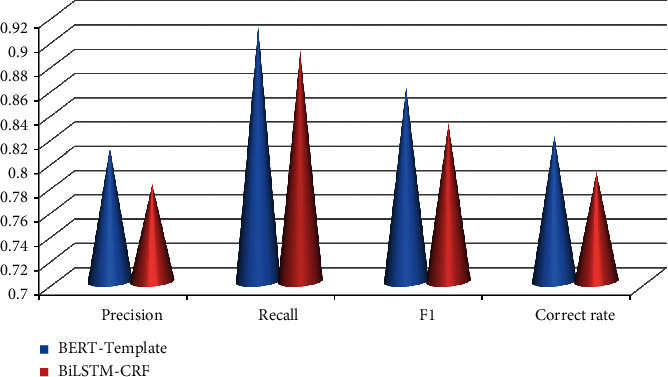
Event classification results on educational experience.

The F1 value of the BERT-Template method is 0.03 and 0.03 higher than that of the BiLSTM-CRF form in the classification of job experience and education experience events, respectively. Overall, the BERT-Template process is better. The event classification results on work experience and educational experience is shown in Figures 4 and 5, respectively.

### 4.5. Evaluation of Character Relationship Graph Construction

**Figure 6:**
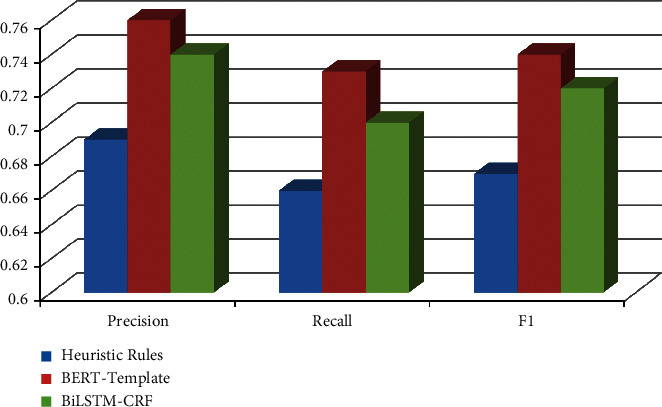
Entity-relationship discovery and extraction result on colleague relationship.

After obtaining the hierarchical personnel templates, the colleague and alumni relationships between the personnel templates are discovered and extracted. The removed personnel relationship and personnel template information are used to build a knowledge graph and store it in the Neo4j graph database.  Figure 6 below graphically shows the entry-relationship discovery and extraction result on colleague relationship.

**Figure 7:**
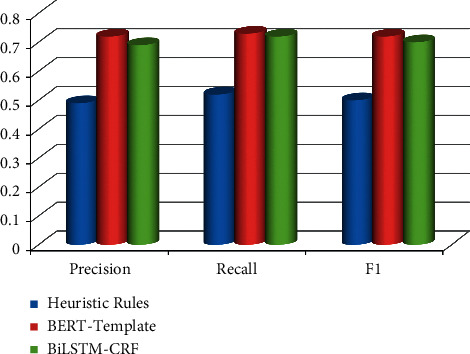
Result analysis.

The hierarchical personnel templates constructed by artificial heuristic rules, BERT-Template method, and BiLSTM-CRF method are used to discover and extract personal relationships, and the artificially created accurate hierarchical personnel templates for personnel relationship discovery and extraction are carried out and compared. The comparison results are shown in Table 5 and Figure 7.

In Table 5, the BERT-Template method is used to discover and extract personal relationships based on the hierarchical personnel templates constructed on the BERT pretraining model. If we compare precision value with *F*_1_ value on the basis of their results, we find that the precision rate increased by 0.07 and 0.23, respectively, and the *F*_1_ value increased by 0.07 and 0.22, respectively. The precision value is more accurate than *F*_1_ value. Compared with the BiLSTM-CRF process, the relationship between colleagues and alumni is accurate compared with the heuristic rule method, in terms of colleague and alumni relations, the precision rate increased by 0.07 and 0.23, respectively, and the *F*_1_ value increased by 0.07 and 0.22, respectively. Overall, the BERT-Template method can achieve better results.

## 5. Conclusions

This study studies the construction method of character relationship graphs in the Chinese financial field and proposes a framework for constructing a character relationship graph in the economic area: person attribute extraction and association problem in unstructured personnel resume text. The current approaches for obtaining information from user resumes do not function well with unorganized user descriptions seen in economic announcements, nor do they work well with papers containing the same users. The BERT model is used to accurately extract personnel attribute entities, the fine-tuned trained BERT model is used to classify event instance vectors, a hierarchical personnel information template is constructed, and the problem of personnel attribute association is solved. Finally, the filled personnel information template is more convenient and accurate. The relationship between people is extracted, and a relationship map of people is built. Experiments show the framework's effectiveness for constructing person relationship graphs in the financial field. The future scope of this research is that the proposed BERT-based framework for constructing a relationship graph of Chinese characters in the financial field is proposed.

This framework relies on manually annotated datasets. The next step is to consider using weakly supervised learning methods to expand the dataset further and reduce manually constructing datasets.

## Figures and Tables

**Table 1:** Description of entity labeling dataset.

Statistical indicators	Quantity
Total number of people entities	1 694
Total characters	597 157
Total number of people attribute entities	86 066
Total number of sentences	14 666

**Table 2:** Description of template dataset.

Statistical indicators	Quantity
Total number of people entities	1 694
Total number of educational experience event instances	2 407
Total number of tenure event instances	26 756
Total number of colleague relationships	52 202
Total alumni relations	2 264

**Table 3:** Experimental results of personnel attribute entity extraction.

Method	Precision	Recall	*F*_1_ value
Heuristic rules	0.811 8	0.776 7	0.793 9
BiLSTM -CRF	0.901 5	0.921 3	0.911 3
BERT	0.921 1	0.934 0	0.927 5

**Table 4:** Event classification results.

Event type	Method	Precision	Recall	F1	Correct rate
Working experiences	BERT model	0.79	0.89	0.88	0.88
	Bi-LSTM-CRF	0.86	0.85	0.83	0.86

Educational experience	BERT-model	0.78	0.89	0.83	0.79
	Bi-LSTM-CRF	0.76	0.86	0.78	0.76

**Table 5:** Result analysis of entity-relationship discovery and extraction.

Relationship type	Method	Precision	Recall	*F*_1_
Colleague relationship	Heuristic model	0.67	0.64	0.62
	BERT model	0.75	0.72	0.72
	BiLSTM-CRF	0.73	0.69	0.68

Alumni relations	Heuristic model	0.52	0.49	0.49
	BERT model	0.68	0.68	0.67
	BiLSTM-CRF	0.65	0.65	0.69

## Data Availability

The data shall be made available on request.
